# Introducing enhanced recovery after surgery in a high-volume orthopaedic hospital: a health technology assessment

**DOI:** 10.1186/s12913-020-05634-3

**Published:** 2020-08-24

**Authors:** Francesco Vanni, Emanuela Foglia, Federico Pennestrì, Lucrezia Ferrario, Giuseppe Banfi

**Affiliations:** 1 IRCCS Orthopedic Institute Galeazzi, Via Riccardo Galeazzi 4, 20161 Milan, Italy; 2 Centre for Health Economics, Social and Health Care Management, LIUC Business School, LIUC – Università Cattaneo, Corso Matteotti 22, 21053 Castellanza, Varese Italy; 3 Vita-Salute San Raffaele University, Via Olgettina 58, 20132 Milan, Italy

**Keywords:** Enhanced recovery after surgery, Joint-arthroplasty, Activity-based analysis, Health technology assessment, EUnetHTA, Cost-effectiveness, Value-based healthcare

## Abstract

**Background:**

The number of patients undergoing joint arthroplasty is increasing worldwide. An Enhanced Recovery After Surgery (ERAS) pathway for hip and knee arthroplasty was introduced in an Italian high-volume research hospital in March 2018.

**Methods:**

The aim of this mixed methods observational study is to perform a health technology assessment (HTA) of the ERAS pathway, considering 938 procedures performed after its implementation, by means of a hospital-based approach derived from the EUnetHTA (European Network for Health Technology Assessment) Core Model. The assessment process is based on dimensions of general relevance, safety, efficacy, effectiveness, economic and financial impact, equity, legal aspects, social and ethical impact, and organizational impact. A narrative review of the literature helped to identify general relevance, safety and efficacy factors, and a set of relevant sub-dimensions submitted to the evaluation of the professionals who use the technology through a 7-item Likert Scale. The economic and financial impact of the ERAS pathway on the hospital budget was supported by quantitative data collected from internal or national registries, employing economic modelling strategies to identify the amount of resources required to implement it.

**Results:**

The relevance of technology under assessment is recognized worldwide. A number of studies show accelerated pathways to dominate conventional approaches on pain reduction, functional recovery, prevention of complications, improvements in tolerability and quality of life, including fragile or vulnerable patients. Qualitative surveys on clinical and functional outcomes confirm most of these benefits. The ERAS pathway is associated with a reduced length of stay in comparison with the Italian hospitalization average for the same procedures, despite the poor spread of the pathway within the country may generate postcode inequalities. The economic analyses show how the resources invested in training activities are largely depreciated by benefits once the technology is permanently introduced, which may generate hospital cost savings of up to 2054,123.44 € per year.

**Conclusions:**

Galeazzi Hospital’s ERAS pathway for hip and knee arthroplasty results preferable to traditional approaches following most of the HTA dimensions, and offers room for further improvement. The more comparable practices are shared, the before this potential improvement can be identified and addressed.

## Background

The spread of chronic musculoskeletal disorders prompted the United Nations, the World Health Organization and 37 countries to proclaim that we are living in the Bone and Joint Decade [1]. The need to provide good quality healthcare at sustainable costs calls for innovative management approaches able to combine effective procedures with efficient allocation of resources.

Improving the value of healthcare became a global priority [2]. At this purpose, the four dimensions of personal, technical, allocative and societal value are being progressively defined by specific indicators of healthcare quality, including clinical, organizational, economic and social implications retrieved from a multidisciplinary and evidence-based health technology assessment (HTA) [3–5].

These efforts are particularly clear in joint arthroplasty (JA), due to high number of procedures delivered worldwide [1]. A number of studies demonstrated how significant improvements in effectiveness and efficiency may not follow from the introduction of a single clinical innovation in the treatment (be it surgical, medical or rehabilitative), but from the way in which the single procedures are related [6–9]. This is the core idea of multidisciplinary accelerated pathways like Fast-Track or Enhanced Recovery After Surgery (ERAS), whose value is maximized by the cooperation between clinical professionals (who offer their expertise on the field) and researchers (who look for best practices and innovations in scientific literature) [10, 11].

An ERAS pathway for hip and knee arthroplasty was introduced in a high-volume research hospital, IRCCS Orthopedic Institute Galeazzi (Milan, Italy), in March 2018. The aim of this study is to perform a multidimensional evaluation of the pathway 9 months after its implementation, in order to i) compare benefits and disadvantages with conventional JA, ii) identify subsequent challenges and room for improvement, iii) offer a potential comparison with similar practices worldwide.

## Methods

An HTA is conducted considering 938 hip and knee JAs performed within the ERAS pathway from March to October 2018. The assessment is based on a hospital version [12] of the EUnetHTA Core Model [13], which includes all the dimensions required by the European framework [13]. The following domains were considered and further divided into appropriate sub-dimensions: i) general relevance of the ERAS pathway (the technology under evaluation); ii) safety; iii) efficacy; iv) economic and financial impact; v) equity; vi) legal aspects; vii) social and ethical impact; viii) organizational impact. Each domain is introduced by the most relevant results selected from a narrative review of the literature, performed by seeking the following keywords on PubMed: “ERAS”, “Enhanced Recovery”, “Fast Track”, “Rapid Recovery”, “Arthroplasty”, “THA”, “TKA”, “orthopaedic”; and including only English language manuscripts.

Qualitative, quantitative, or mixed assessments of the technology are performed according to the type of evidence investigated in the single domain. Qualitative assessments of the ERAS pathway are performed through the administration of a 7-item Likert scale validated questionnaire [14] to the 38 healthcare professionals who employ the technology daily (orthopaedic surgeons, anaesthesiologists, physiotherapists, nurses, and supporting staff), who rated their experience from − 3 to + 3 (the higher the preference, the better the technology). 71% (27) of these professionals answered the questionnaire. According to the EUNetHTA Core Model, the sample is enough to perform a comparable analysis with other providers [13]. Turning subjective preferences into numerical entities, the Likert scale allows to compare the conventional approach with the ERAS pathway, based on the direct evaluation of the healthcare professionals who employ these technologies daily, and calculate the respective average scores assigned on indicators of interest.

Quantitative assessment is performed by gathering comparable data from 1) internal and/or national registries, such as length of hospital stay (LOS) in acute ward or in rehabilitation setting, and 2) economic analysis tools recommended by the literature. Any comment on the results is postponed to the discussion section. The general relevance of the technology is introduced by a narrative literature review and described more in detail by the comparison between conventional treatment and ERAS pathway, as currently performed within the same hospital.

Focusing on statistical analysis, data were analysed considering descriptive statistics, frequencies, and distributions. After having tested the normal distribution of all the variables investigated (− 1 < Skewness< 1 and − 1 < Kurtosis< 1), independent sample t-tests were used to describe the existence of statistically significant differences between the presence or the absence of ERAS pathway, within the qualitative perceptions.

All analyses were conducted with a significance level of 0.05. Results are expressed as average value ± standard error.

## Results

### General relevance

A healthcare technology is relevant when comparable with another existing procedure to treat a significant epidemiological need. JA is considered the best solution against osteoarthritis and rheumatoid arthritis when conservative treatment is not effective, with increasing worldwide patients undergoing surgery primarily due to aging, overweight and trauma [15, 16].

In Italy, a dedicated National Register (Registro Italiano Artroprotesi, RIAP) was introduced in 2006 in order to collect epidemiological and demographic information associated with patient characteristics from the entire country. According to the last report [17], joint arthroplasties are increasing by 4.1% per year with a major prevalence of hip (56.3%) and knee (38.6%) procedures, following arthrosis as the main diagnosis; the remaining 3.9, 0.3 and 0.9% surgeries are related to shoulder, ankle and other joints. In 2015, respectively 96.1 and 96.6% of hip and knee JAs were financed by the Italian National Health Service (Servizio Sanitario Nazionale, SSN), and the trend is expected to increase. For these reasons, any technology able to ensure quality, and improve sustainability is a relevant innovation which could benefit patients, hospitals and funders.

A high variety in patient characteristics, surgeon preferences, logistical arrangements, and regional healthcare financing makes it difficult to compare the approaches adopted by different providers, both in Italy and other European countries [18]. Moreover, more than half of Italian providers are represented by low-volume healthcare institutions, which are generally disadvantaged in implementing effective accelerated pathways [19, 20]. This is why most of the comparisons between conventional treatment and accelerated pathways are performed within the same healthcare supplier or through before-after observational studies [21–23].

The contents of conventional treatment and ERAS pathway as currently provided by Galeazzi Hospital are reported in Table 1.

**Table 1:** Galeazzi Hospital - Conventional treatment and ERAS pathway

	Conventional (up to February, 2018).	ERAS (from March, 2018).
Preoperative.	Preoperative visit with orthopaedic surgeon and anaesthesiologist (diagnostic exams included). Informed consent.	Standard preoperative visit. 1-h preoperative group education with a physiotherapist and a nurse, in which details on the pathway are given to the patient in order to facilitate engagement. The patient is given life-style advice about the risks of smoking, alcohol and bad nutrition in order to maximize postoperative recovery. The physical therapist describes the muscle strengthening exercises to be performed before surgery and the information which the patient needs to get in advance (crutches, walkers, elastic stockings, etc.). The social conditions of the patients are taken into evaluation in order to verify the presence or not of a caregiver. In order to reduce preoperative fasting as much as possible, the patient is given a Carbohydrate loading (2 maltodextrins flasks) with relative instructions for consumption (1 at midnight before day of surgery, 1 at 6.00 AM the day of surgery). Blood management (identification and correction of anaemia). Informed consent. Pre-emptive oral analgesia.
Intraoperative.	Surgery according to the surgeon’s choice. Sub-arachnoid anaesthesia. Drains and catheterization.	Tranexamic acid is administered before incision in order to reduce perioperative bleeding. Tissue-sparing surgery according to the surgeon’s choice. Selective sub-arachnoid anaesthesia in order to maintain vital parameters as stable as possible. Adductor canal block for total knee arthroplasty (TKA). Local Infiltration Analgesia (LIA) before surgical suture, if needed, depending on the evaluation of the anaesthesiologist. Possibly no drains and catheterization.
Postoperative.	Pain management according to the surgeon’s choice. Mobilization and physiotherapy from 1 day after surgery, once a day, for half an hour. Pharmacological treatment in case of nausea and vomiting, followed by light dinner or fasting.	Multimodal pain management according to the surgeon’s choice, including if possible opioid-sparing analgesia. Postoperative nausea and vomiting prophylaxis. Feeding 3 h after surgery, with tea and rusks. Mobilization 4–6 h after surgery, assisted by 2 physioterapists, once safety conditions are guaranteed by the anaesthesiologist. Assisted walking with crutches. Light dinner. Pharmacological treatment of nausea and vomiting if needed. Gastric protection and intestinal prokinetics treatments in order to prevent paralytic ileus. Two physiotherapy sessions from 1 day after surgery, half an hour each.
Average Length of Stay (LOS).	Average 5.2 days in the acute ward, then a) If the patient does not reach a sufficient level of autonomy, or is not supported by family caregiving: transfer to the rehabilitation unit. Average LOS for rehabilitation: 20 days. b) If the patient reaches a sufficient level of autonomy to face home discharge: direct home discharge.	a) If the patient is affected by clinical and social conditions of fragility resulting from the preoperative assessment; or by risk factors and complications that emerged later: 3 days LOS in acute orthopaedic ward + internal rehabilitation depending on the need. b) If the recovery proceeds normally: up to 5 days LOS in acute orthopaedic ward + direct home discharge. Functional exams are performed depending on the surgeon’s choice.
Perioperative.	No audit between the professionals involved in the treatment. Dedicated nurses. Non-dedicated physical therapists (turnover between different wards and procedures).	Internal audit (ward data analysis and problem solving) every 4 months. Dedicated acute ward, physiotherapists and nurses.

### Safety

Faster recovery must not preclude safe recovery. Up to 52% of reduced hospital LOS was obtained without a significant increase in readmissions [24–27], except for old, frail, or psychiatric patients affected by clinical comorbidities could not bear the efforts of enhanced recovery. This occurence will be discussed the domain of equity. Reduced readmissions, complications, transfusions and mortality have been found by comparative studies, reviews and meta-analyses from 1 week to 10 years after surgery [5–35]. An increased risk of urinary tract infections was resolved by gradually abandoning the use of bladder catheters [36]. The incidence of postoperative delirium and cognitive dysfunction was reduced by the introduction of opioid-sparing analgesia [37, 38].

The 938 procedures under assessment included hip and knee, primary and revision, unilateral and bilateral, total and unicompartmental, simultaneous and staged JAs, offering an exhaustive sample of surgical procedures. Eight different surgical teams accepted to adopt the ERAS pathway for all the patients undergoing hip or knee arthroplasty in their unit, without selecting for patient characteristics or type of surgery. Follow-up visits are generally performed after 6 weeks, 3 months, 6 months and 1 year, depending on the surgeon’s decision, the evolution of recovery and patient characteristics. Since all the procedures were performed between March and October 2018, at the moment of writing it is impossible to collect data on long-run complications, readmissions, and patient experiences of pain.

**Fig. 1:**
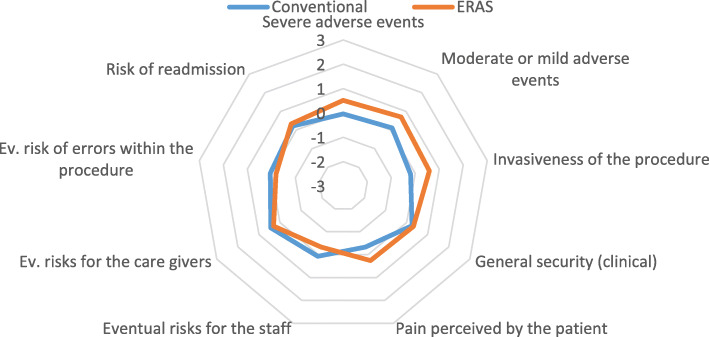
Safety domain

The lack of comparative data required preliminary estimations of safety to be carried out by means of a qualitative questionnaire, completed by the 27 healthcare professionals currently employed in the ERAS pathway. According to the EUnetHTA Core Model, they evaluated 9 safety indicators which were largely retrieved from the before-mentioned literature review (Fig. 1).

ERAS pathway dominates conventional treatment on patient safety, reporting a slight improvement in the mean rate (0.29 ± 0.011 vs 0.07 ± 0.006). General security, tolerability, pain, and risk of readmission are the items in which the technology dominates with a difference of more than 0.50 units, calculated by the mean preferences expressed by the professionals through the 7-item Likert Scale. On the contrary, the technology seems not to be equally safe in terms of errors within the procedure, which may trigger risks to the healthcare providers and caregivers which do not comply with a certain innovation introduced within the pathway. However, the mean difference perceived in comparison with the conventional procedure is under 0.50 units.

### Efficacy

Efficacy is different from effectiveness. Efficacy is retrieved from the literature as a parameter of how a certain treatment performs in different settings and/or different patients. Effectiveness is a real-world indicator of how the same treatment performs in a specific setting on specific patients. Then, the efficacy of a certain intervention (“can it work?”) is a preliminary condition to test the effectiveness of the same intervention under usual clinical circumstances (“does it work in practice”?) [12, 39].

A number of studies support the efficacy of accelerated pathways. A retrospective comparative study between conventional and accelerated total knee revisions (TKRs) resulted in faster postoperative recovery for those patients who underwent the latter [6]. According to a randomized controlled trial, accelerated TKA resulted in significantly lower knee pain scores and improved functional outcomes 7 days after surgery [32]. An evidence-based review on blood management in Total Joint Arthroplasty (TJA) underlined how accelerated pathways provide useful room for perioperative planning and prediction, generating additional value in comparison to a conventional protocol [9]. A prospective cohort study reported improved function, quality of life, reduced pain, and better Patient-Related Outcome Measurements (PROMs) after accelerated total hip arthroplasty (THA) [40]. Unicompartmental Knee Arthroplasty (UKA) performed under an accelerated pathway including minimally invasive surgery, discharge the same day of surgery, and no inpatient physiotherapy reported earlier improvements in knee motion in comparison to a conventional, more invasive approach, equal safety provided [41]. Similar findings were reported by a retrospective observational study on TJAs in a Veteran setting, which concludes how these protocols are essential to support the “transition into an era of value-based arthroplasty” [42].

Accelerated perioperative care and rehabilitation after hip and knee arthroplasty demonstrated good results under different settings and circumstances [42], which provides preliminary evidence of efficiency to investigate the specific degree of effectiveness.

### Effectiveness

Specific clinical and logistical indicators to assess the effectiveness of accelerated pathways have been identified in maximum 3 days of acute ward LOS, improved patient autonomy and function, positive PROMS, and survivorship after surgery [30, 38].

In the absence of long-term data on patients who underwent surgery in the ERAS pathway, a preliminary estimation of the effectiveness of the technology was performed in three steps.

1. Comparing the average patient LOS in Galeazzi acute and rehabilitation wards (collecting data from the internal registry) with the average LOS in Italian hospitals (collected from RIAP, year 2017), to perform a hip or knee arthroplasty (Table 2). Since RIAP does not allow to distinguish between acute ward and rehabilitation, the overall LOS was considered for comparison. Demographic characteristics such as age and sex are also reported (Table 3).
2. Administering a qualitative questionnaire on 5 indicators of effectiveness [13] retrieved from the before-mentioned literature overview. As long as subjective experiences like pain and PROMS are not available at the time of writing, they are replaced by feedback from individual patients as reported by the staff.
3. Reporting the number of postoperative deaths based on legal complaints.

**Table 2:** Overall days of LOS, ERAS Galeazzi vs. Italian standard

Site of surgery.		Italy (RIAP 2017).	ERAS Galeazzi (2018).
Hip.	Primary.	8.1	4.2 ± 0.27
	Revision.	13.3	4.2 ± 0.56
Knee.	Primary.	7.6	4.4 ± 0.16
	Revision.	9.7	4.6 ± 0.61

**Table 3:** Mean age and sex prevalence, ERAS Galeazzi vs Italian Standard [F (female), M (male)]

	Italy (RIAP 2017).		ERAS Galeazzi (2018).
	Hip Arthroplasties.	Knee Arthroplasties.	Distinction not available.
Mean age.	F 74.7; M 69.2	F 70.7; M 69.5	F 70.1; M 66.7
Sex prevalence (%).	F 61.2; M 38.8	F 67.9; M 32.1	F 61.9; M 38.1

**Fig. 2:**
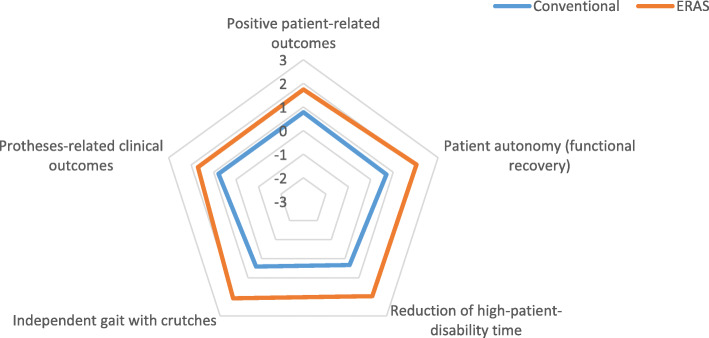
Effectiveness domain

The average LOS associated with the ERAS pathway is reduced in comparison to Italian hospitals. Patients undergoing surgery within the pathway are slightly younger and more likely to be males (Fig. 2).

According to the qualitative survey, ERAS dominates the conventional treatment in each of the five indicators, maintaining a difference of more than 0.5 units of preference (1.90 ± 0.076 vs 0.60 ± 0.095). No patients died during hospitalization or after discharge.

### Economic and financial impact

Value is given by healthcare outcomes per dollar spent [43]. Accelerated pathways demonstrated to reduce the cost of arthroplasty up to 1 year-postoperatively [44], resulting cost-effective in different settings [42, 45].

The economic and financial impact of the ERAS pathway was evaluated by adopting the following approaches [46–48].

1. An Activity Based Costing (ABC) [46], in order to compare the cost of the technology under assessment with the previous cost of conventional treatment in the same hospital.
2. A Cost-Effective Analysis (CEA), by means of an Incremental Cost-Effectiveness Ratio (ICER), in order to define the cost-effectiveness ratios generated by conventional treatment and ERAS technology, based on the hospital internal data [48].
3. A Budget Impact Analysis (BIA), calculated from the number of patients treated within the ERAS pathway, multiplied by the cost of the average process, assuming the hospital perspective [47], and considering a 12-month time range, as if the new technology entirely replaced the previous treatment.

All costs are expressed in Euros.

#### Activity based costing analysis (ABC)

The cost of the technology is calculated by the sum of the human and technical resources employed along the process, assuming the hospital perspective (Table 4).

**Table 4:** Activity Based Costing Analysis (€)

	Preoperative (per patient).	Intraoperative (per patient).	Surgical ward (per day of hospitalization).	Rehab. (per day of hospitalization).
ERAS Activity Based Costing Analysis.				
Human Resources.	42.00	327.80	104.00	153.90
Technologies and equipment.	0	24.21	16.00	16.00
Drugs, consumables, prostheses.	7.00	1410.64	65.52	65.52
Other.	0	85.92	32.62	32.62
Total per each phase.	49.00	1848.52	218.14	268.75
**Total per process.**	**2383.75**			
Conventional treatment Activity Based Costing Analysis (€).				
Human Resources.	24.00	327.80	94.10	153.90
Technologies and equipment.	0	24.21	16.00	16.00
Drugs, consumables, prostheses.	0	1410.64	65.52	65.52
Other.	0	85.92	32.62	32.62
Total per each phase.	24.00	1848.52	208,24	268.75
**Total per process.**	**2349.56**			

Assuming the hospital perspective, the ERAS pathway costs € 34,19 more than the conventional pathway, where the human resources employed in the preoperative phase represent the main difference.

#### Cost-effectiveness analysis (CEA)

Qualitative indicators of effectiveness do not allow rigorous comparisons between similar pathways, and postoperative survivorship is a short-term indicator of effectiveness. Then, an evidence-based comparison between ERAS pathway and conventional treatment can be performed by adopting hospital LOS as a preliminary indicator of the latter (Table 5).

**Table 5:** ICER (€ per day)

	Conventional treatment.	ERAS treatment.
Cost (€).	2349.56	2383.75
Average LOS in Orthopaedic Surgery (in days).	5.2	4.6
**ICER.**	**−56.98**	

The CEA resulted in −56.98 ICER score, which underlines the greater effectiveness of the ERAS pathway against a moderate economic investment. According to the above, considering an average economic value of a single hospitalization day in Orthopaedic surgery equal to € 218.14, the reduction in LOS will be translated in an economic saving per patient equal to € 130.88 for the hospitalization in Orthopaedic surgery.

#### Budget impact analysis (BIA)

Guaranteed the effectiveness and safety of each treatment, the ERAS pathway is associated with reduced patient LOS and increased discharge directly at home. Considered the average reimbursement of € 2244.77 per patient rehabilitation charged by the hospital to the SSN, the technology under assessment generates significant savings in terms of public healthcare expenditure.

Table 6 compares the amount of public funds spent by the SSN to cover hip and knee arthroplasties under conventional treatment (year 2017) and ERAS pathway.

**Table 6:** Public healthcare expenditure savings following reduced LOS and increased discharge to home (€)

	2017 (conventional).	March–October 2018 (ERAS).
Number of patients.	1271	938
Average LOS in Orthopaedic Surgery.	5.2 days	4.6 days
Patients % discharged directly to home.	12.00% (153)	36.14% (339)
Patients % undergoing inpatient rehabilitation.	88.00% (1118)	63.86% (599)
Average rehabilitation cost per patient.	€ 2244.77	2244.77
**Total expenditure.**	**€ 2,510,730.35**	**€ 1,821,991.37**

Assuming the public funder perspective, the analysis shows how the introduction of the new technology saved € 688,738.93 to the SSN in less than 1 year, following from reduced LOS and no need for inpatient rehabilitation. However, this evaluation is performed by comparing 12 months of conventional treatment against 9 months of accelerated pathway. In order to uniform the evaluation, consider the scenario in which the ERAS pathway entirely replaced conventional treatment, 1) assuming the same number of patients (1271) from 1 year to the next; 2) including in detail the following items of healthcare expenditure (Table 7):

1. the cost of admission to orthopaedic surgery (medical costs);
2. the cost of the surgical activity (major surgical procedures);
3. the cost of the rehabilitation activities.

**Table 7:** Total public healthcare expenditure and ERAS-generated saving (€)

	2017 (conventional)	1-year ERAS
Number of patients treated annually for hip and knee JA.	1271	1271 (projection of 100% ERAS pathway replacement rate).
Average cost of hospitalization to orthopaedic surgery for 1 patient (total costs per medical procedure: € 218.14, multiplied by average LOS for conventional procedure: 9.7 days; and ERAS pathway: 4.6).	2110.50	1036.24
Total expenditure for 1 year.	2,682,445.50	1,317,061.64
Average cost of surgery for 1 patient (total costs to perform a surgical procedure).	1848.52	1848.52
Total expenditure for 1 year.	2349,468.92	2349,468.92
Average cost or rehabilitation for 1 patient.	2244.77	2244.77
Number of patients rehabilitated.	1118 (88% of patients admitted to the conventional treatment).	813 (64% of patients admitted to the ERAS pathway, calculated out of the projection).
Annual total health care expenditure.	7,542,644.77	5,488,521.33
**ERAS pathway-induced savings in case of entire replacement** **of the conventional treatment.**	**/**	**2054, 123.44**

If the ERAS pathway entirely replaced conventional treatment on the same number of patients in 1 year, the technology would generate savings of € 2054,123.44 per year (−27%) in comparison to the baseline scenario. Thus, the ERAS accelerated pathway is more affordable than the conventional treatment.

### Equity

The dimension of equity can be distinguished on a macro and a micro level [49, 50].

On the macro level, equity is a matter of allocative justice. Achieving the same outcomes with fewer resources (technical value) allows the public funder to free up financial resources which can be reinvested in treating other morbidities, or a higher number of arthritic patients, with a potential reduction in hospital waiting lists, providing better access to free and quality health care (personal, allocative and societal value). In this sense, the economic and financial assessments support equity of the ERAS pathway on a macro level.

On the micro level, equity is a matter of healthcare accessibility to vulnerable categories of patients. Caution must be kept in case of patients who find it difficult to bear early mobilization and intensive rehabilitation, be them psychiatric, old, frail, or affected by other clinical morbidities (i.e. chronic) or conditions of risk. Despite accelerated pathways show improved accessibility to protected categories of patients [9, 51, 52], their safety remains controversial [53–58].

**Fig. 3:**
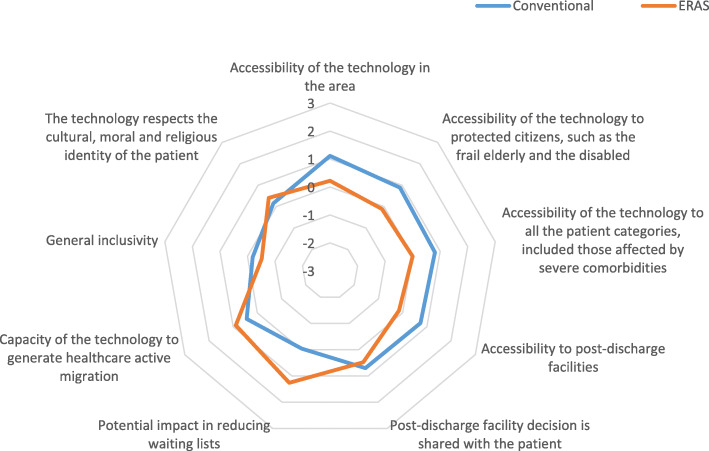
Equity domain

A qualitative investigation of the equity domain is conducted through 9 indicators retrieved from EUnetHTA Core Model [13] (accessibility of the technology at local level, impact on hospital waiting lists, general inclusivity and ability of the technology to respect the cultural, moral and religious identity of patients) and the before-mentioned literature overview (clinical comorbidity and social frailty) (Fig. 3).

The conventional treatment (average value = 0.49 ± 0.0135) seemed slightly preferable than the ERAS pathway (average value = 0.33 ± 0.0172) when analysed in light of the equity domain.

### Legal, social, and ethical impact

The impact of the ERAS pathway from the perspective of society is here declined in terms of i) the human and economic burden removed from the patient and/or his/her family or caregivers, ii) the estimated productivity loss caused by the attendance of the healthcare environment, iii) the need to support the spread and safety of the technology with adequate legal procedures and market regulation.

**Fig. 4:**
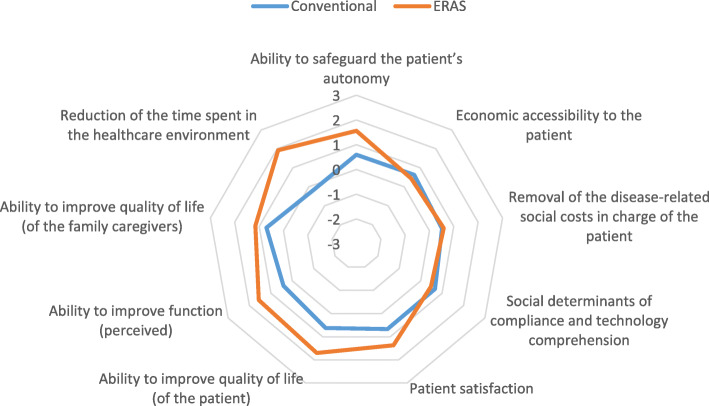
Burden removed by the technology

**Fig. 5:**
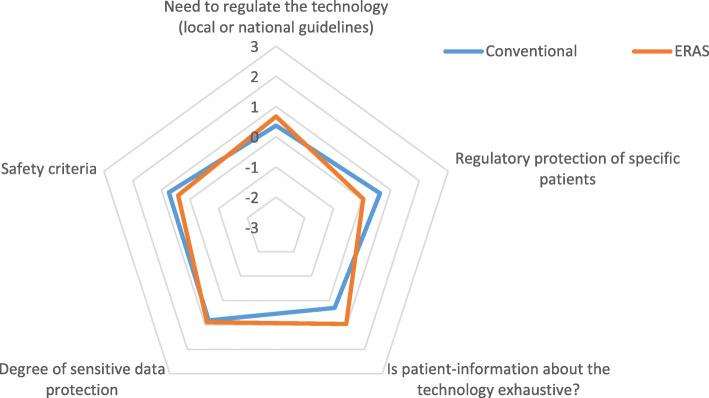
Legal domain

Points i) and iii) had been evaluated by the administration of a 7-item Likert Scale qualitative questionnaire to the healthcare professionals answering the questionnaire (Figs. 4 and 5); point ii) had been evaluated by calculating the amount of days spent by the patient in the healthcare environment, including hospital admission and rehabilitation process (Table 6).

#### Human and social burden removed by the technology (Fig. 4)

ERAS pathway dominated the conventional treatment (average value: 1.17 ± 0.139 vs 0.59 ± 0.084) in light of the burden removed by the technology, with the exception of two indicators: economic accessibility to the patient (ability to pay), and social determinants of compliance and technology understanding (i.e. patient language and patient degree of education, health literacy, and consequent ability to comply with the pathway).

#### Productivity loss

The perceived reduction of time spent by the patient in the healthcare environment (Fig. 4) is confirmed by the internal hospital register. Table 8 shows how patients who underwent surgery under the ERAS pathway spent on average 4.39 days less in the healthcare environment (surgery and eventual inpatient rehabilitation), in comparison to patients who underwent surgery under the conventional treatment.

**Table 8:** Time spent in the healthcare environment

	2017 (conventional)	2018 March–October (ERAS)
Number of patients.	1271	938
% of which undergoing inpatient rehabilitation.	88	63.86
Average LOS in surgery ward.	5.2 days	4.5 days
Average days for rehabilitation.	12.1	10.9
Total days spent by total patients in the surgical ward.	6609.2	4221
Total days spent by total patients for rehabilitation.	13,533.6	6529.2
Total days spent in the healthcare environment.	20,142.8	10,750.2
Average days spent *inside* the healthcare environment for each patient (regardless of rehabilitation or not).	15.85	11.46
**Variation in the average days spent** ***inside*** **the healthcare environment.**	**4.39**	

Multiplying the average days spent outside the healthcare environment by the number of patients who underwent surgery under the ERAS pathway, in 9 months, the technology saved 4115 days of hospital attendance in comparison to the conventional treatment.

#### Legal procedures and regulation (Fig. 5)

Figure 5 shows how ERAS pathway and conventional treatment reach almost equivalent scores (average value: 0.59 ± 0.079 vs 0.58 ± 0.085) with regards to the legal domain.

### Organizational impact

The sustainability of innovation is given not only from a profitable ratio between costs and benefits, but also from the amount of investments required to replace the previous technology. The potential investments required to implement the ERAS pathway are here described in terms of:

- The percentage of professionals (right column) agreeing or not to perform a certain investment (left column), including potential specific suggestions (Table 9);
- The cost of 30-h specific training to all the professionals who employ the technology (Table 10).

**Table 9:** Quantitative analysis of the investments required according to ERAS professionals

Items.	Additional investments required (average).
Additional staff.	78% favourable. On average, 2.8 more nurses, 2.8 more physical therapists, 1.9 more social health operators, 1 case manager, 1 internist, 1 nutritionist, and 1 dedicated physiatrist were requested.
Training.	Specific courses for every professional involved. 1-h training to the patients and their informal caregivers by a nurse and a physical-therapists when in operation (88.9%).
Communications/meetings.	Permanent periodic audit (100%).
Spaces and furnishings.	More space (62.9%), average 43 m^2^. New furniture: a dedicated gym (18.5%), more PCs, desks and chairs (14.8%), electric beds (14.8%), showers (3.7%).
Machinery and equipment.	Additional Continuous Passive Motion machines (18.5%); intraoperative traction beds, ultrasounds, Patient-Controlled Analgesic (PCA) pumps, telemedicine (3.7%).
Management tools and software.	33.3% professionals favourable (inter-operational patient/ward registers, communication between hospital, general practitioner and rehabilitation facilities).

**Table 10:** Cost of 30 h specific training to all the professionals employed by the technology

Professional.	Hourly cost (€).	Hours.	Units needed in ERAS.	Loss of production (€).
Orthopaedic surgeon.	32.12	30	15	14,454.00
Anaesthesiologist.	32.12	30	10	9636.00
Physical therapist.	15.87	30	4	1904.40
Nurse.	16.18	30	15	7281.00
Supporting staff.	13.39	30	3	1205.00
**Total cost.**				**34,480.50**

The benefits of telemedicine [59], permanent audit [7, 22, 34] and management technologies [19] are confirmed in the literature. However, adequate professional training courses must be dedicated to all the professionals involved in their use. To estimate the costs of 30-h training activity in the hospital under consideration, the cost per hour of each professional is multiplied by the number of professionals needed in the ERAS pathway, and by the number of training hours.

Introducing a 30-h training activity would require to the hospital an additional investment of € 34,480.50. Considering the € 2054,123.44 estimated savings generated by the introduction of the ERAS pathway in 1 year, the economic investment in training activities should be largely outweighed by the economic benefits induced by the technology.

## Discussion

### General relevance

JA procedures are increasingly performed to treat hip and knee arthritis each year, supported by a number of international studies designed to improve their clinical tolerability and financial sustainability. The ERAS pathway introduced in Galeazzi Hospital, which aims to improve the treatment by investing on preoperative planning and perioperative integration of the single medical and surgical performance, can therefore be considered relevant.

### Safety

According to the perception of the professionals involved in the ERAS pathway, the new technology dominates the conventional treatment in terms of general clinical security, tolerability by the patient, reduced pain, and risk of adverse events or readmissions. Improved scores could be explained by the multidisciplinary commitment required by the technology, better communication among the professionals, progress in pain management, and accurate evaluation of the single patient [6, 22, 60, 61]. Precautions against urinary trait infections and postoperative delirium, as suggested from the literature [36–38], are adopted within the pathway (i.e. bladder catheter removal and opioid-sparing analgesia whenever possible). On the contrary, the introduction of the new technology may trigger more risks for the professionals who operate within the pathway, since the high level of cooperation which is required may be undermined by perioperative fragmentation. However, specific training and increasing experience are likely to overcome this limit over time. A limit of the present study is the impossibility to measure safety issues from a formal internal registry. However, if considerable adverse events and complications did occur during the pathway, these events would probably have affected the staff ratings, which were performed under conditions of anonymity to avoid bias. Further research may include PROMs in the assessment of this dimension, in order to consider the direct patient’s perspective, improve patient-oriented care and further reduce potential bias.

### Efficacy

Increasing literature shows accelerated pathways to dominate more conventional approaches in terms of reduced pain scores, earlier functional recovery, improved quality of life, and better PROMs. This dimension was not supported by a systematic review of the evidence. However, the primary interest of the manuscript is to perform a validated HTA on a specific hospital-based preliminary experience, then the dimension of effectiveness is expected to provide more valuable and substantial information - in comparison to the dimension of efficacy - to share with the clinical and academic community.

### Effectiveness

The technology reduced LOS in comparison to the conventional treatment performed in Italian hospitals. Improved clinical and functional outcomes were reported by the professionals employed within the pathway. The limited time available for data collection and the subsequent impossibility to include PROMs suggest caution in the interpretation of these results. However, longer-lasting clinical studies based on similar outcomes, PROMs included, from 90 days to 10 years after surgery, support positive expectations towards the technology [21, 33, 35, 36, 62–64]. Patients undergoing surgery within the pathway are slightly younger and more likely to be males, although the impossibility to distinguish between hip and knee procedures makes it difficult to investigate the potential determinants of this variation.

### Economic and financial impact

The ABC analysis has estimated that the ERAS pathway costs to the hospital € 34.19 more than the previous, conventional treatment. The incremented cost is mainly due to more investments in human resources at the preoperative phase, in order to provide patient assessment and education. Despite the benefits of pre-operative education for hip and knee replacement are controversial [65, 66], current evidence supports the effectiveness of an adequate patient assessment over the perioperative process [9, 51, 67], which is corroborated by the improved outcomes perceived by the professionals involved in the ERAS pathway. This finding may support the hypothesis that accelerated pathways do not differ from conventional treatment in medical content or surgical technique, but rather from the way in which the perioperative process is planned and integrated, which is probably what makes ERAS pathway a valuable innovation [9].

A moderate additional investment at the preoperative phase is outweighed by a reduction in hospital LOS, which - once the safety of the procedures is maintained - reduces in turn the time spent by the patient in the healthcare environment, generates significant cost savings to the funder, and potentially reduce waiting lists in order to provide a higher number of treatments. The cost-effectiveness of the ERAS pathway is therefore supported by a CEA. It may be argued that reduced hospital LOS is a poor indicator of the effectiveness of a medical treatment, for increased risk of readmissions and increased cost-shifting to the patient may follow. With regard to cost-shifting, ERAS pathway outpatient rehabilitation is provided by Galeazzi Hospital and funded by SSN, without any additional cost to the patient. With regard to readmissions, the lack of long-term data about patient recovery is actually a limit of the present assessment; however, waiting for those data to be available, evidence from longer-lasting studies and positive impressions by the involved professionals support the cost-effectiveness of the ERAS pathway.

Assuming the hospital perspective, projections of one-year implementation of the ERAS pathway on the same number of patients treated in 2017 under the conventional pathway (as if the technology entirely replaced the previous treatment), generated an estimated saving of € 2054,123.44 per year. Based on these analytical tools, the ERAS pathway is expected to generate positive economic and financial impact. Although the estimations are based on projections on the same number of months and patients, they offer a perspective of the significant economic benefits that the systematic adoption of the ERAS pathway should generate on the long term.

### Equity

According to the professionals involved in the ERAS pathway, the conventional treatment dominates the new technology on 6 out of 9 indicators. Lower outcomes are reported in relation to the accessibility of the technology in the area, the accessibility to post-discharge facilities, and the possibility to choose post-discharge facility together with patient. These results are probably due to the poor spread of the technology outside the hospital: if outpatient rehabilitation requires a specific approach which is compatible with an accelerated pathway, at least in terms of perioperative coordination among professionals, patients who live far from the hospital will find it difficult to obtain adequate support, even more if they are elderly, disabled or frail [6, 39, 49, 50]. Lower outcomes are reported also in relation to the general inclusivity of the technology, the accessibility to protected citizens and the accessibility to all patient categories. These results are probably due to the patients who may find it difficult to bear the psychophysical efforts requested by accelerated pathways. However, several studies demonstrate accelerated pathways’ improved eligibility to patients older than 85 years or affected by psychiatric disorders or severe comorbidities [54, 68–71], although some pathological limits may remain difficult to overcome [58, 72]. Among the 3 indicators in which the ERAS pathway dominates the conventional treatment, the capacity of the technology to generate active healthcare migration confirms its poor spread outside the hospital. The more the technology proves effective and cost-effective, the more it is likely to be adopted by other health care suppliers and financed by SSN, which would improve the pathway’s accessibility and the connection between facilities. These considerations highlight the significant room for improvement offered by the introduction of the ERAS pathway on the dimension of equity, which are expected to improve realistically over time. Further research may include PROMs in the assessment of this dimension, in order to reduce potential bias. Meanwhile, those patients who are not eligible for the pathway may still be enrolled in the conventional treatment, then they are not left untreated.

### Legal, social, and ethical impact

The functional impairment caused by joint pain is a human and economic burden, since quality of life, autonomy and productivity are critically threatened. JA is considered the best solution against osteoarthritis and rheumatoid arthritis when a conservative treatment is not effective. Accelerated pathways present the additional advantage to reduce the time spent within the healthcare environment. However, the new technology seems to present some issues in terms of economic accessibility to the patient and social determinants of compliance. The problem of economic accessibility may occur to patients who need to undergo outpatient rehabilitation compatible with the ERAS pathway. Since such a specific approach may not be provided or funded by the SSN throughout the entire country, the patients who live far from Galeazzi Hospital are likely to pay the service out-of-pocket. The inability of a patient to access equal healthcare based on the area of the country in which he/she was born, or lives, is known in the literature as post-code lottery [50]. Similar to the issues discussed with regard to the domain of equity, this difficulty is likely to be overcome by the increasing spread of accelerated pathways.

With regard to the social determinants of compliance, patients respond to clinical instructions when they are able to understand them, and believe that they will be helpful in solving their problems [73]. Multidisciplinary pathways may be more difficult to understand by socially frail patients, be it for cognitive decline, isolation, language, ethnicity, or education level. Despite patients often appreciate a reduction in hospital LOS [74], they are also worried about being left alone to fragmented healthcare [75, 76]. In the short term, part of these issues can be resolved by proper patient or family education, which is provided within the ERAS pathway, and it is perceived as exhaustive by the professionals involved (“Is patient-information about the technology exhaustive?”). In order to take the best advantage from patient education [65, 74, 77], information must be clear, accessible, and the more patient-centred as possible [78]. In the long term, improved coordination between healthcare institutions and dedicated training to the professionals involved may also play a positive role.

With regard to the subdomain of productivity loss, the professionals perceive the ERAS pathway to reduce the time spent in the healthcare environment, as a consequence of reduced hospital LOS and increased discharge directly at home. With regard to the subdomain of legal procedures and regulation, both the conventional treatment and the ERAS pathway need improvements in terms of care providers’ compliance to guidelines and patients’ data protection. The spread of the new technology, supported by a permanent audit, a multidisciplinary commitment between professionals, clinical feedback, and the share of best practice among researchers, are likely to support this need [6, 46, 48]. Based on these considerations, the ERAS pathway offers room for improvement in terms of legal, social and ethical impact.

### Organizational impact

The economic resources invested in support of professional training activities were estimated to be largely outweighed by benefits once the technology is permanently introduced. Most of the expenditure is likely to occur during the phase of implementation. The more cooperation between the professionals involved in the pathway, hospital governance and administration, the better identification of the organizational issues to be addressed, included the enrollment of dedicated staff. Therefore, the organizational impact of the ERAS pathway is probably sustainable and cost-effective in the long-term.

This HTA involved the commitment of a researcher (not on the payroll) and the support from a few administrative employees (already on the payroll) to access to the data. Therefore, it did not require any additional cost to the hospital. Conversely, clinical, organizational and economic benefits are expected from the improvement of the technology.

## Conclusion

The aim of an HTA is to choose the technology associated to the best “added value” among different alternatives. Value can be conceived in several ways, which include different outcomes, various time ranges and several perspectives. The ERAS pathway under assessment meets most of the considered outcomes and presents considerable room for further improvement. The more experiences are shared and compared, the before this room for improvement can be addressed.

## Data Availability

Quantitative data were gathered from internal or national registries. Qualitative data were gathered by administering a 7-item Likert scale to 27 the care providers involved in the technology. The National Registry is mentioned in the references. Internal registries and qualitative questionnaires are reserved.

## Abbreviations

ABC: Activity Basted Costing
BIA: Budget Impact Analysis
CEA: Cost-Effectiveness Analysis
ERAS: Enhanced Recovery After Surgery
HTA: Health Technology Assessment
ICER: Incremental Cost-Effectiveness Ratio
JA: Joint Arthroplasty
LOS: Length Of Stay
PROMs: Patient-Related Outcomes
RIAP: Registro Italiano Artroprotesi
SSN: Servizio Sanitario Nazionale
THA: Total Hip Arthroplasty
TJA: Total Joint Arthroplasty
TKA: Total Knee arthroplasty
UKA: Unicompartmental Knee Arthroplasty
